# FGF signal is not required for hepatoblast differentiation of human iPS cells

**DOI:** 10.1038/s41598-019-40305-2

**Published:** 2019-03-06

**Authors:** Yukiko Toba, Ayumi Kiso, Souichiro Nakamae, Fuminori Sakurai, Kazuo Takayama, Hiroyuki Mizuguchi

**Affiliations:** 1 Laboratory of Biochemistry and Molecular Biology, Graduate School of Pharmaceutical Sciences, Osaka University, Osaka, 565-0871 Japan; 2 Laboratory of Hepatocyte Regulation, National Institutes of Biomedical Innovation, Health and Nutrition, Osaka, 567-0085 Japan; 3 PRESTO, Japan Science and Technology Agency, Saitama, 332-0012 Japan; 4 Global Center for Medical Engineering and Informatics, Osaka University, Osaka, 565-0871 Japan

## Abstract

Human induced pluripotent stem cell-derived hepatocyte-like cells are expected to be utilized in pharmaceutical research and regenerative medicine. In general, human induced pluripotent stem (iPS) cells are differentiated into hepatocyte-like cells through definitive endoderm cells and hepatoblast-like cells using various growth factors that are essential for liver development. Although recombinant bone morphogenetic proteins (BMPs) and fibroblast growth factors (FGFs) are widely used in the hepatoblast differentiation, hepatoblast differentiation process has not been fully modified. In this study, we examined the roles of BMPs and FGFs in the hepatoblast differentiation from human iPS cells. Surprisingly, the gene expression levels of hepatoblast markers were upregulated by the removal of FGFs. In addition, the percentages of hepatoblast markers-positive cells were increased by the removal of FGFs. Furthermore, the hepatocyte differentiation potency was also significantly increased by the removal of FGFs. To examine whether FGF signals are completely unnecessary for the hepatoblast differentiation, the expression levels of endogenous FGF ligands and receptors were examined. The definitive endoderm cells highly expressed the FGF ligand, FGF2, and the FGF receptor, FGFR1. To examine the role of endogenous FGF signals, an FGFR inhibitor was treated during the hepatoblast differentiation. The hepatoblast differentiation was promoted by using FGFR inhibitor, suggesting that endogenous FGF signals are also unnecessary for the hepatoblast differentiation. In conclusion, we found that FGF signals are not essential for hepatoblast differentiation. We believe that our finding will be useful for generating functional hepatocyte-like cells for medical applications.

## Introduction

Human induced pluripotent stem (iPS) cell-derived hepatocyte-like cells (HLCs) are expected to be utilized in pharmaceutical research and regenerative medicine. It is essential to generate functional and homogenous HLCs from human iPS cells for such applications. Human iPS cells can be differentiated into HLCs through definitive endoderm cells and hepatoblast-like cells. In general, most of the hepatocyte differentiation methods use several growth factors that play important roles in mouse, Xenopus, and zebrafish liver development. Activin A is widely used for the definitive endoderm differentiation. Hepatocyte growth factor (HGF) and oncostatin M (OsM) are widely used for the hepatocyte maturation process from hepatoblast-like cells. However, the growth factors, which are used in the hepatoblast differentiation, vary among the different hepatocyte differentiation protocols^1–6^. Therefore, we expect that optimizing the hepatoblast differentiation protocol will be essential for the generation of functional and homogenous HLCs.

Previous embryological studies of mouse, Xenopus, and zebrafish liver development have shown that bone morphogenetic protein (BMP) and fibroblast growth factor (FGF) signals are necessary for liver bud formation^7,8^. Jung *et al*. have reported that Fgf1 and Fgf2, which are both secreted from the cardiac mesoderm, were required for the differentiation into alfa-fetoprotein and transthyretin-positive tissue by using mouse embryo tissue culture system^7^. Rossi *et al*. have also demonstrated that Bmp2, Bmp4 and Bmp7, which are all secreted from the cardiac mesoderm and the septum transversum mesenchyme, were necessary for the induction of albumin gene expression^8^. Consistently, the expression levels of hematopoietically expressed homeobox and prospero-related homeobox 1 in hepatoblasts were significantly reduced when Bmp receptors or Fgf receptors were blocked by using dominant-negative forms of Bmp or Fgf receptors^9^. From these studies, Bmp and Fgf signals have been considered to be essential also for the hepatoblast differentiation from embryonic stem (ES) cells and iPS cells. In fact, Gouon-Evans *et al*. have reported that Bmp4 and Fgf2 were necessary for the hepatoblast differentiation from “mouse” ES cell-derived definitive endoderm cells^10^. However, it has not been fully examined whether BMP and FGF signals are also essential for the hepatoblast differentiation from “human” iPS cell-derived definitive endoderm cells.

As well as Bmp and Fgf ligands, Bmp and Fgf receptors are greatly related to early embryonic development including hepatogenesis^11,12^. Jung *et al*. have reported that Fgfr1- and Fgfr2-positive “mouse” definitive endoderm develops into afp- and alb-positive liver bud by Fgf1 or Fgf2 treatment^7^. Kishigami *et al*. and de Sousa Lopes *et al*. have reported that BmpR1A-, BmpR2-, and ActR2A-positive “mouse” definitive endoderm develops into liver bud in response to Bmp treatment^13,14^. However, the expression profiles of FGF and BMP receptors and their functions have not been fully examined in human iPS cell-derived definitive endoderm cells.

In this study, we performed screening of human recombinant BMP and FGF to optimize the hepatoblast differentiation method from human iPS cells. The hepatocyte differentiation potency of hepatoblast-like cells generated by the optimal differentiation method was also examined. In addition, we examined the expression profile and functions of FGF ligands and receptors in the hepatoblast differentiation.

## Results

### Human recombinant FGFs were not required for hepatoblast differentiation from definitive endoderm cells

**Figure 1:**
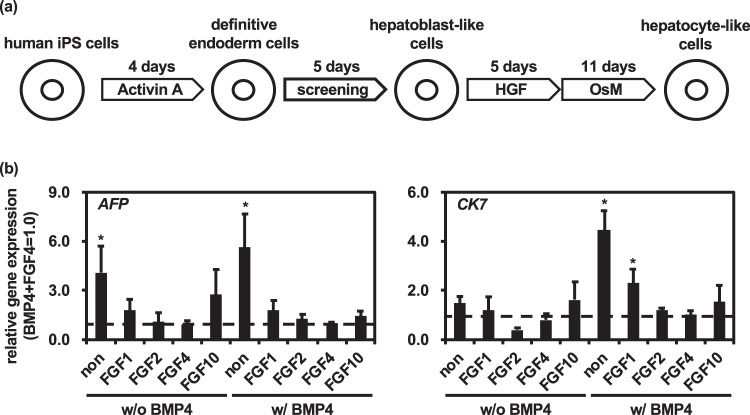
The effect of various FGFs on hepatoblast differentiation. (**a**) The procedure for screening is presented schematically. Details of the hepatocyte differentiation procedure are described in the Materials and Methods section. The cell culture reagents used for screening were 20 ng/mL BMP4, 30 ng/ml FGF1, 10 ng/ml FGF2, 20 ng/ml FGF4, and 20 ng/ml FGF10. (**b**) The gene expression levels of hepatoblast markers (*AFP* and *CK7*) were examined by real-time RT-PCR. The gene expression levels of FGF4 and BMP4-treated cells were taken as 1.0. Mean ± SD (*n* = 3). Statistical significance was evaluated by one-way ANOVA followed by Dunnett’s post-hoc tests (*p < 0.05, compared with “FGF4 and BMP4-treated cells”).

We first examined the definitive endoderm differentiation efficiency by FACS analysis. The percentage of definitive endoderm marker (C-X-C chemokine receptor (CXCR) type 4 (CXCR4))-positive cells was temporally increased during the definitive endoderm differentiation (Fig. S1). At day 4 of differentiation, the percentage of CXCR4-positive cells was approximately 95%. To examine the effects of BMP and FGF on the hepatoblast differentiation from human iPS cell-derived definitive endoderm cells, we performed screening of BMP4 and FGF1/2/4/10 (Fig. 1a). At day 9 of differentiation, the gene expression levels of hepatoblast markers (*alpha-fetoprotein (AFP)* and *cytokeratin (CK) 7*) were examined by real-time RT-PCR. In the presence of BMP4, the gene expression levels of hepatoblast markers in the hepatoblast-like cells differentiated without FGFs were higher than those in the hepatoblast-like cells differentiated with FGFs (Fig. 1b). Furthermore, in the presence of BMP4, the gene expression levels of the pluripotent marker (*octamer-binding transcription factor (OCT) 3/4*) and the definitive endoderm marker (*Sry-related HMG-box gene (SOX) 17*) in hepatoblast-like cells differentiated without FGFs were decreased as compared with those of hepatoblast-like cells differentiated with FGF1/2/4 (Fig. S2). These results suggest that definitive endoderm cells could be efficiently differentiated into hepatoblast-like cells by the removal of FGFs. In the following experiments, we further characterized three groups that were respectively treated with the protocols: “BMP4 + FGF4 = a conventional hepatoblast differentiation protocol”, “without BMP4 and FGF4 = a hepatoblast differentiation protocol using neither BMP4 nor FGF4”, and “BMP4 only = a hepatoblast differentiation protocol using BMP4, but not FGF4”.

### Hepatoblast-like cells were efficiently generated without using FGFs

**Figure 2:**
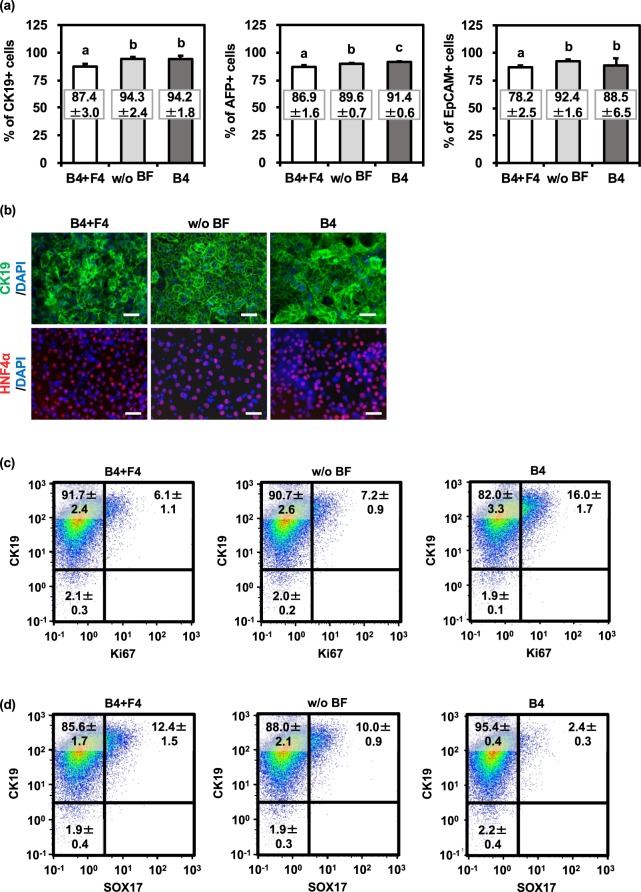
Hepatoblast differentiation was promoted by FGF removal from differentiation medium. Human iPS cells were differentiated into definitive endoderm cells. The definitive endoderm cells were treated with 20 ng/mL BMP4 (B4), with 20 ng/mL BMP4 + 20 ng/mL FGF4 (B4 + F4), or without BMP4 and FGF4 (w/o BF). (**a**) The percentages of hepatoblast markers (CK19, AFP, and EpCAM)-positive cells were examined by FACS analysis. (**b**) The protein expression levels of hepatoblast markers (CK19 (green), HNF4a (red)) were examined by immunocytochemical analysis. Nuclei were counterstained with DAPI (blue). The scale bars represent 50 µm. (**c**) The percentages of hepatoblast marker (EpCAM) and cell proliferation marker (Ki67)-positive cells were examined by FACS analysis. (**d**) The percentages of hepatoblast marker (CK19) and definitive endoderm marker (SOX17)-positive cells were examined by FACS analysis. Mean ± SD (*n* = 3). Statistical significance was evaluated by one-way ANOVA followed by Tukey’s post-hoc tests to compare all groups. Groups that do not share the same letter are significantly different from each other (*p < 0.05).

Hepatoblast-like cells were generated from human iPS cell-derived definitive endoderm cells in the three conditions described above: “BMP4 + FGF4 (B4 + F4)”, “without BMP4 and FGF4 (w/o BF)”, and “BMP4 only (B4)”. To examine hepatoblast differentiation efficiency, the percentages of hepatoblast markers (CK19, AFP, and epithelial cell adhesion molecule (EpCAM))-positive cells were measured by FACS analysis. As compared with hepatoblast-like cells differentiated with BMP4 + FGF4, the percentages of hepatoblast marker-positive cells were increased in the hepatoblast-like cells differentiated without BF or with BMP4 (Fig. 2a). In addition, almost all of the hepatoblast-like cells were positive for hepatoblast markers (CK19 and hepatocyte nuclear factor (HNF) 4α) at day 9 of differentiation (Fig. 2b). These results suggest that hepatoblast differentiation could be promoted by removing FGF4. As well as human iPS cell line, YOW-iPS cells, similar results could be obtained using the human ES cell line, KhES-3 (Fig. S3). To examine the effect of the removal of FGF4 on the hepatoblast proliferation potency^15^, the percentage of cell proliferation marker (Ki67)-positive cells and cell viability in the hepatoblast-like cells were examined by FACS analysis (Fig. 2c) and WST-8 assay (Fig. S4), respectively. The percentage of CK19 and Ki67 double-positive cells in the hepatoblast-like cells differentiated with BMP4 (16.0%) was higher than that of the hepatoblast-like cells differentiated with BMP4 + FGF4 (6.1%) or without BF (7.2%) (Fig. 2c). At day 9 of differentiation, there was the increasing tendency of the cell viability in the hepatoblast-like cells differentiated with BMP4 (115%) compared to that in the hepatoblast-like cells differentiated with BMP4 + FGF4 (100%) or without BF (68.9%) (Fig. S4). These results suggest that the hepatoblast proliferation potency was not inhibited by removal of FGF4. To quantify residual definitive endoderm cells at day 9 of differentiation, the percentage of definitive endoderm marker (SOX17)-positive cells in the hepatoblast-like cells was measured by FACS analysis (Fig. 2d). The percentage of CK19 and SOX17 double-positive cells in the hepatoblast-like cells differentiated with BMP4 (2.4%) was lower than that in the hepatoblast-like cells differentiated with BMP4 + FGF4 (12.4%) or without BF (10.0%). Furthermore, the percentage of CK19 single-positive cells was increased by removing FGF4. These results suggest that hepatoblast-like cells differentiated with BMP4 were the most homogenous among these three groups. Thus, we next examined the hepatocyte differentiation potency of hepatoblast-like cells generated with BMP4.

### FGF-non-treated hepatoblast-like cells retain high hepatocyte differentiation potency

**Figure 3:**
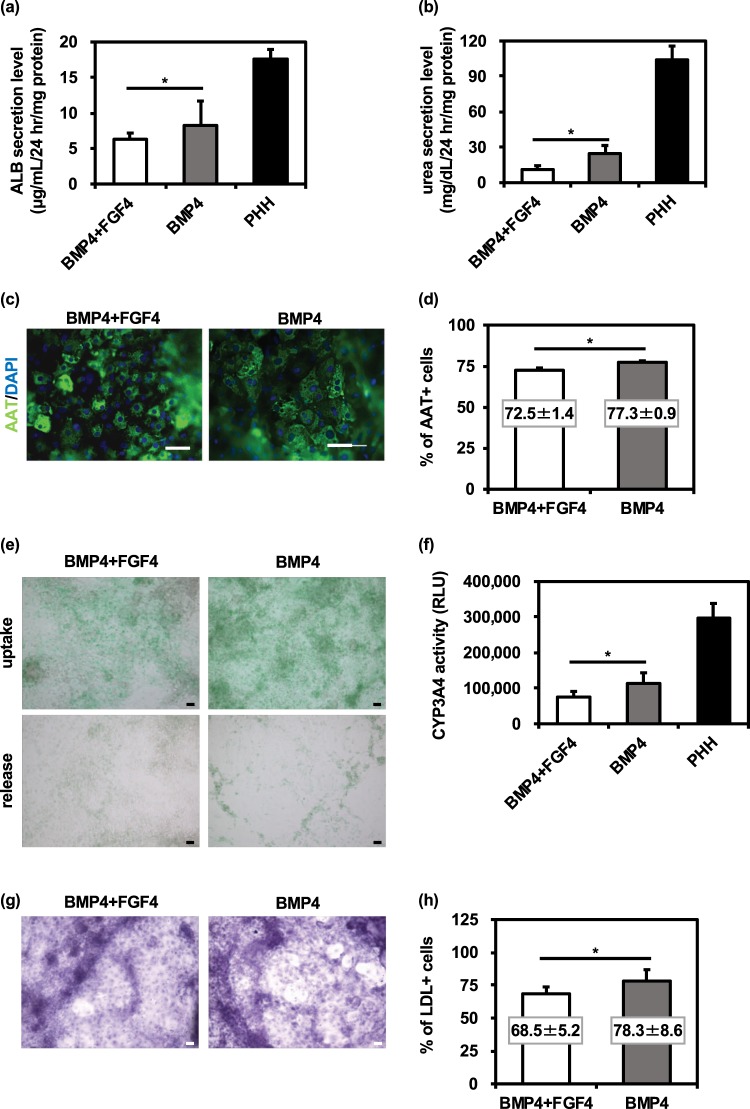
FGF-untreated hepatoblast-like cells retain high hepatocyte differentiation potency. Human iPS cells were differentiated into definitive endoderm cells. The definitive endoderm cells were treated with 20 ng/mL BMP4 or 20 ng/mL BMP4 + 20 ng/mL FGF4 for 5 days. The hepatoblast-like cells were differentiated into HLCs. (**a**,**b**) The ALB (**a**) and urea (**b**) secretion capacities were examined. PHH: Primary human hepatocyte (PHH) cultured for 48 hr after plating. (**c**) The protein expression levels of hepatocyte marker, AAT (green), were examined by immunocytochemical analysis. Nuclei were counterstained with DAPI (blue). The scale bars represent 50 µm. (**d**) The percentage of hepatocyte marker (AAT)-positive cells was measured by FACS analysis. (**e**) The HLCs were examined for their ability to take up Indocyanin Green (ICG) (upper panel) and release it 16 hr thereafter (lower panel). The scale bars represent 50 µm. **(f**) The CYP3A4 activity was examined by using a P450-Glo™ CYP3A4 Assay Kit. (**g**) The glycogen storage of the HLCs was assessed by Periodic Acid-Schiff (PAS) staining. The glycogen storage is indicated by pink or dark red-purple cytoplasms. The scale bars represent 50 µm. (**h**) The HLCs were cultured with medium containing Alexa-Flour 488-labeled LDL for 1 hr, and flow cytometry analysis was performed. The percentage of LDL-positive cells was measured by flow cytometry. Mean ± SD (*n* = 3). Statistical significance was evaluated by unpaired two-tail Student’s *t* test (*p < 0.05, compared with “BMP4 + FGF4”).

As shown in Fig. 1a, HLCs were generated from the hepatoblast-like cells differentiated with BMP4 + FGF4 or BMP4. At day 25 of differentiation, the ALB and urea secretion capacities were examined. The amount of both ALB and urea secretion in the HLCs generated from the hepatoblast-like cells differentiated with BMP4 was higher than that in HLCs generated from the hepatoblast-like cells differentiated with BMP4 + FGF4 (Fig. 3a,b, respectively). At day 25 of differentiation, most of the HLCs were positive for the hepatocyte marker (alpha-1 antitrypsin (AAT)) (Fig. 3c). We also confirmed that the percentage of AAT-positive cells in HLCs differentiated with BMP4 was significantly higher than that in HLCs differentiated with BMP4 + FGF4 (Fig. 3d). As well as the human iPS cell line, YOW-iPS cells, similar results could be obtained using the human ES cell line, KhES-3 (Fig. S5). In addition, we examined whether hepatoblast-like cells generated with BMP4 could differentiate into functional HLCs. The HLCs generated from hepatoblast-like cells differentiated with BMP4 had higher ability to uptake Indocyanine green (ICG) as compared with HLCs generated from hepatoblast-like cells differentiated with BMP4 + FGF4 (Fig. 3e). The CYP3A4 activity level of the HLCs generated from hepatoblast-like cells treated with BMP4 was also higher than that of the HLCs generated from hepatoblast-like cells treated BMP4 + FGF4 (Fig. 3f). We confirmed the ability of the HLCs to store glycogen using the PAS staining assay (Fig. 3g). The ability of low-density lipoprotein (LDL) uptake was examined by FACS analysis. The percentage of LDL-positive cells in the HLCs differentiated with BMP4 was significantly higher than that in HLCs differentiated with BMP4 + FGF4 (Fig. 3h). From these results, the hepatoblast-like cells differentiated with BMP4 showed higher hepatocyte differentiation potency as compared with the hepatoblast-like cells differentiated with BMP4 + FGF4.

### Endogenous FGF signals were not required for hepatoblast differentiation from definitive endoderm cells

**Figure 4:**
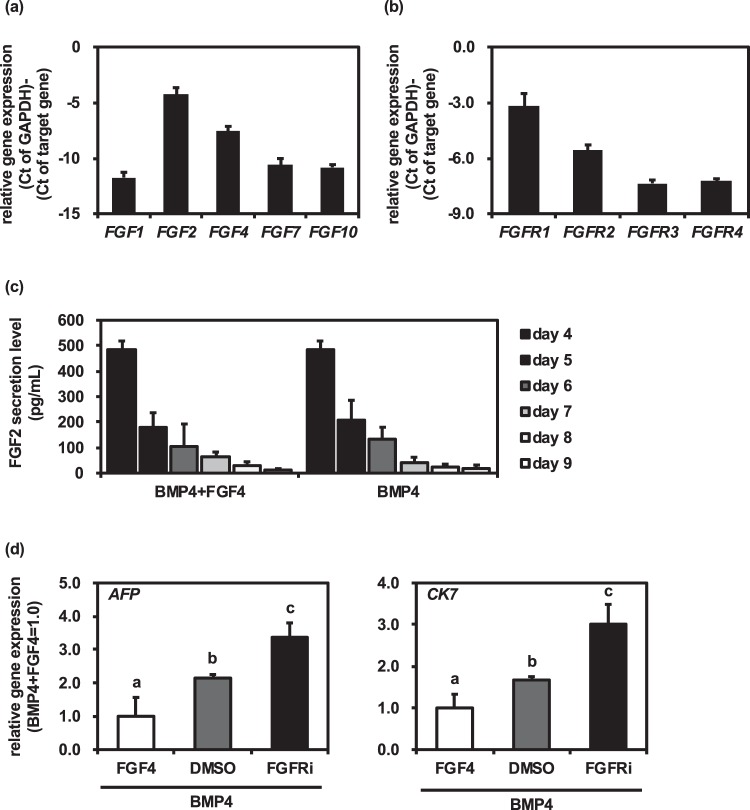
FGF inhibitor promoted hepatoblast differentiation. Human iPS cells were differentiated into definitive endoderm cells. The definitive endoderm cells were treated with 20 ng/mL BMP4 or 20 ng/mL BMP4 + 20 ng/mL FGF4 for 5 days. (**a,b**) At day 4 of the differentiation, the gene expression levels of the FGF ligands (*FGF1*, *FGF2*, *FGF4*, *FGF7*, and *FGF10*) (**a**) and receptors (*FGFR1*, *FGFR2*, *FGFR3*, and *FGFR4*) (**b**) were measured the definitive endoderm cells by real-time RT-PCR. (**c**) The temporal FGF2 secretion levels were examined by ELISA analysis. (**d**) Human iPS cells were differentiated into definitive endoderm cells. The definitive endoderm cells were treated with 10 nM FGF receptor inhibitor, FIIN 1 hydrochloride (FGFRi), for 5 days. The gene expression levels of hepatoblast markers (*AFP* and *CK7*) were examined by real-time RT-PCR. The gene expression levels in BMP4 + FGF4-treated cells were taken as 1.0. Mean ± SD (*n* = 3). Statistical significance was evaluated by one-way ANOVA followed by Tukey’s post-hoc tests to compare all groups. Groups that do not share the same letter are significantly different from each other (*p* < 0.05).

To examine whether FGF signals are completely unnecessary for the hepatoblast differentiation from human iPS cell-derived definitive endoderm cells, we first examined the gene expression profiles of FGF ligands and receptors in human iPS cell-derived definitive endoderm cells. The gene expression levels of *FGF2* and *FGFR1* were the highest among the various FGF ligands and receptors, respectively (Fig. 4a,b). During the hepatoblast differentiation, the FGF2 secretion level was measured by ELISA. The amount of FGF2 secretion was gradually decreased during the hepatoblast differentiation (Fig. 4c). It is known that FGFR1 is one of the major receptors of FGF2^16,17^. Therefore, we expected that the endogenous FGF2 might regulate the hepatoblast differentiation from definitive endoderm cells in an autocrine manner. To investigate whether the inhibition of FGF receptors affects the hepatoblast differentiation, an FGFR inhibitor, FIIN 1 hydrochloride, was used. At day 9 of differentiation, the gene expression levels of hepatoblast markers (*AFP* and *CK7*) in the FGFR inhibitor-treated cells were upregulated (Fig. 4d). These results suggest that endogenous FGF signals prevented the hepatoblast differentiation from human iPS cell-derived definitive endoderm cells.

## Discussion

In this study, we optimized the hepatoblast differentiation method from human iPS cell-derived definitive endoderm cells. Hepatoblast-like cells were efficiently generated by the removal of FGF signals. Furthermore, the hepatoblast-like cells differentiated with BMP4 (without FGF4) showed higher hepatocyte differentiation potency as compared with the hepatoblast-like cells generated by the conventional differentiation method using both BMP4 and FGF4.

We screened human recombinant FGFs in order to optimize the hepatoblast differentiation protocol, and found that the removal of FGFs was effective for highly efficient hepatoblast differentiation (Figs 1 and 2). Ameri *et al*. have previously optimized the concentration of FGF2 during the hepatoblast differentiation from human ES cell-derived definitive endoderm cells. As compared with a high concentration of FGF2 (>16 ng/ml), a low concentration of FGF2 (4 ng/mL) induced higher ALB expression levels. In addition, high FGF2 concentration promoted differentiation toward pancreatic and intestinal lineages but not hepatic lineage^18^. It has also been reported that treatment with an FgfR inhibitor (SU5402) did not alter the protein expression levels of hepatoblast markers (hnf4α and prox1) in the posterior liver bud by using the analysis of mouse embryo^19^. These previous findings are consistent with our findings in this study, suggesting that an FGF signal is not required for the hepatoblast differentiation. On the other hand, Twaroski *et al*. have reported that the gene expression levels of *FOXA2*, *HEX*, and *HNF4*α in human iPS cell-derived definitive endoderm cells were decreased by treatment with an FGFR inhibitor in the presence of BMP4 and FGF2^20^. However, we speculate that the hepatoblast differentiation potency could not be fully examined in their study because such hepatoblast markers (FOXA2, HEX, and HNF4α) are anterior definitive endoderm markers^21,22^. Therefore, we consider that this discrepancy between our findings and those of Twaroski *et al*. might be due to the difference in the markers which were used in the expression analysis.

The amount of FGF2 secretion in the definitive endoderm cells (485 pg/ml) was significantly higher than that in the hepatoblast-like cells (15 pg/ml) (Fig. 4c). This result suggests that endogenous FGF signals are necessary for the maintenance of definitive endoderm cells but not necessary for hepatoblast differentiation and its maintenance. Because it is known that FGFs activate the MAPK pathway^23,24^, the MAPK pathway might be activated in the definitive endoderm cells, but not in the hepatoblast-like cells. The function of the MAPK pathway in definitive endoderm cells and hepatoblast-like cells would be elucidated by using MEK inhibitors in the hepatoblast differentiation process.

## Conclusions

In this study, we succeeded in performing efficient hepatoblast differentiation without use of FGFs. Compared with the conventional hepatoblast differentiation methods using both BMP and FGF, the cost for hepatoblast differentiation using our method would likely be lower. In addition, residual definitive endoderm cells were significantly decreased by our approach, suggesting that it might be possible to generate safer HLCs with low risk for teratoma formation. We believe that human iPS cell-derived HLCs generated by our differentiation method could be useful for pharmaceutical research and regenerative medicine.

## Materials and Methods

### Culture reagents

The cell culture reagents used for hepatocyte differentiation were 20 ng/mL BMP4, 30 ng/ml FGF1, 10 ng/ml FGF2, 20 ng/ml FGF4, and 20 ng/ml FGF10. FGF2 was purchased from Katayama Chemical Industries. All other reagents were from R&D Systems. The FGF receptor inhibitor, FIIN 1 hydrochloride, was purchased from R&D Systems. During the hepatocyte differentiation, the human iPS cell-derivatives were cultured with the medium containing 10 nM FIIN 1 hydrochloride.

### Human iPS cells

The human iPS cell lines, YOW-iPS cells^5^, generated from primary human hepatocytes were maintained on a feeder layer of mitomycin C-treated MEF with ReproStem medium supplemented with 10 ng/ml FGF2^25^. Human iPS cell line, YOW-iPS cells, was used in Figs 1–4, S1, S2 and S4.

### Human ES cells

The KhES-3 line (Kyoto University) was maintained on a feeder layer of mitomycin C-treated mouse embryonic fibroblasts (Merck Millipore) in ReproStem medium (ReproCELL) supplemented with 5 ng/ml FGF2 (Katayama Kagaku Kogyo). KhES-3 was used following the Guidelines for Derivation and Utilization of Human Embryonic Stem Cells of the Ministry of Education, Culture, Sports, Science and Technology of Japan and the study was approved by the Independent Ethics Committee. The human ES cell line, KhES-3, was used in Figs S3 and S5.

### Hepatocyte differentiation

For each human ES/iPS cell line used in hepatocyte differentiation, all differentiated cells were constantly removed by manual collection with a pipette. Before the initiation of hepatocyte differentiation, human iPS cells were dissociated into clumps by using dispase (Roche) and plated onto BD Matrigel Basement Membrane Matrix Growth Factor Reduced (BD Biosciences). These cells were cultured in the MEF-conditioned medium for 3–4 days. The differentiation protocol for the induction of definitive endoderm cells, hepatoblast-like cells, and HLCs was based on our previous reports with some modifications^5^. Briefly, in the definitive endoderm differentiation, human iPS cells were cultured for 4 days in Wnt3A-expressing L cell (ATCC, CRL2647)-conditioned RPMI1640 medium (Sigma), which contained 100 ng/ml Activin A (R&D Systems), 1 × GlutaMAX, 0.2% fetal bovine serum (FBS), and 1 × B27 Supplement Minus Vitamin A (Thermo Fisher Scientific). For the induction of hepatoblast-like cells, the definitive endoderm cells were cultured for 5 days in RPMI1640 medium (Sigma) which contained 20 ng/ml BMP4 (R&D Systems), 20 ng/ml FGF4 (R&D Systems), 1 × GlutaMAX, and 1 × B27 Supplement Minus Vitamin A. To perform the hepatocyte differentiation, the hepatoblast-like cells were cultured for 5 days in RPMI1640 medium (Sigma), which contained 20 ng/ml HGF, 1 × GlutaMAX, and 1 × B27 Supplement Minus Vitamin A. Finally, the cells were cultured for 11 days in Hepatocyte Culture Medium (HCM, Lonza) without EGF but with 20 ng/ml oncostatin M (OsM).

### Real-time RT-PCR

Total RNA was isolated from human iPS cells and their derivatives using ISOGENE (NIPPON GENE). cDNA was synthesized using 500 ng of total RNA with a Superscript VILO cDNA synthesis kit (Thermo Fisher Scientific). Real-time RT-PCR was performed with SYBR Green PCR Master Mix (Applied Biosystems) using a StepOnePlus real-time PCR system (Applied Biosystems). The relative quantitation of target mRNA levels was performed by using the 2^−ΔΔCT^ method. The values were normalized by those of the housekeeping gene, *glyceraldehyde 3-phosphate dehydrogenase* (*GAPDH*). PCR primer sequences (described in Table S1) were obtained from PrimerBank (https://pga.mgh.harvard.edu/primerbank/).

### ALB and urea secretion

The culture supernatants, which were incubated for 24 hr after fresh medium was added, were collected and analyzed by Enzyme-Linked Immuno Sorbent Assay (ELISA) to determine their levels of ALB secretion. A Human Albumin ELISA Quantitation Set was purchased from Bethyl Laboratories. ELISA was performed according to the manufacturer’s instructions. The amount of ALB secretion was calculated according to each standard followed by normalization to the protein content per well.

The culture supernatants, which were incubated for 24 hr after fresh medium was added, were collected and analyzed for the amount of urea production. Urea measurement kits were purchased from BioAssay Systems. The experiment was performed according to the manufacturer’s instructions. The amount of urea secretion was calculated according to each standard followed by normalization to the protein content per well.

### FGF2 secretion

The culture supernatants, which were incubated for 24 hr after fresh medium was added, were collected and analyzed by ELISA to determine their levels of FGF2 secretion. The Human FGF basic Quantikine ELISA Kit was purchased from R&D Systems, and ELISA was performed according to the manufacturer’s instructions.

### Immunocytochemistry

To perform the immunocytochemistry, the human ES/iPS cell-derived cells were fixed with 4% paraformaldehyde (PFA) in PBS for 10 min. After blocking the cells with PBS containing 10% FBS, 1% bovine serum albumin (BSA), and 1% Triton X-100 for 45 min, the cells were incubated with a primary antibody (described in Table S2) at 4 °C overnight, and then with a secondary antibody (described in Table S2) at room temperature for 1 hr.

### Fluorescence-activated cell sorting (FACS) analysis

Single-cell suspensions of the human ES/iPS cell-derived cells were fixed with 4% PFA at 4 °C for 10 min, and then incubated with the primary antibody (described in Table S3), followed by the secondary antibody (described in Table S3). Analysis was performed on an MACSQuant Analyzer (Miltenyi Biotec) and FlowJo software (FlowJo LLC, http://www.flowjo.com/).

### Cellular uptake and excretion of ICG

ICG (Sigma) was dissolved in DMSO at 100 mg/ml, then added to a culture medium of the HLCs to a final concentration of 1 mg/ml at day 25 of differentiation. After incubation at 37 °C for 60 min, the medium with ICG was discarded and the cells were washed with phosphate-buffered saline. The cellular uptake of ICG was then examined by microscopy. Phosphate-buffered saline was then replaced by the culture medium and the cells were incubated at 37 °C for 16 hr. The excretion of ICG was examined by microscopy.

### Periodic Acid-Schiff assay for glycogen

The HLCs were fixed with 4% paraformaldehyde and stained using a Periodic Acid-Schiff staining system (Sigma) at day 25 of differentiation according to the manufacturer’s instructions.

### Uptake of LDL

The HLCs were cultured with medium containing Alexa-488-labeled LDL (Thermo Fisher Scientific) for 1 hr, and then the cells that could uptake LDL were assessed by flow cytometry.

### Primary human hepatocytes (PHH)

Three lots of cryopreserved human hepatocytes (lots YOW, OHO, and FCL; Veritas) were used. The data for the primary human hepatocytes are the average values of the three lots. The vials of hepatocytes were rapidly thawed in a shaking water bath at 37 °C; the contents of each vial were emptied into prewarmed Cryopreserved Hepatocyte Recovery Medium (CHRM; Thermo Fisher Scientific) and the suspension was centrifuged at 900 rpm for 10 min at room temperature. The hepatocytes were seeded at 1.25 × 10^5^ cells/cm^2^ in HCM containing 10% fetal calf serum (FCS) (GIBCO) onto type I collagen-coated 12-well plates. The medium was replaced with HCM 6 hr after seeding. The hepatocytes, which were cultured 48 hr after plating the cells, were used in the experiments.

## Supplementary information

Supplemental file
