# Supplementary material for: FGF signal is not required for hepatoblast differentiation of human iPS cells
Source: Sci Rep. 2019 Mar 6;9:3713. doi: 10.1038/s41598-019-40305-2 (PMC6403225; doi:10.1038/s41598-019-40305-2)
Supplement: Supplementary file 1 — Supplemental file [file 41598_2019_40305_MOESM1_ESM.docx]

**Title**

FGF signal is not required for hepatoblast differentiation of human iPS cells

**Authors**

Yukiko Toba, Ayumi Kiso, Souichiro Nakamae, Fuminori Sakurai, Kazuo Takayama, Hiroyuki Mizuguchi

**INVENTORY OF SUPPLEMENTAL INFORMATION**

**Supplemental Figures (Figures S1-S5)**

**Supplemental Tables (Tables S1-S3)**

**Supplemental figures**

**Figure S1 The efficiency of the definitive endoderm differentiation from human iPS cells**

Human iPS cells (YOW-iPS cells, day 0) were treated with Activin A for 4 days. The percentage of definitive endoderm marker (CXCR4)-positive cells was examined by FACS analysis. Mean ± SD.

**Figure S2** **Hepatoblast differentiation of human iPS cells was promoted by FGF removal from differentiation medium**

Human iPS cells (YOW-iPS cells) were differentiated into the definitive endoderm cells. The definitive endoderm cells were differentiated into hepatoblast-like cells as described in **Figure 1a**. The gene expression levels of pluripotent marker (*OCT3/4*) and definitive endoderm marker (*SOX17*) were examined by real-time RT-PCR. The gene expression levels in BMP4+FGF4-treated cells were taken as 1.0. Mean ± SD. Statistical significance was evaluated by one-way ANOVA followed by Dunnett’s post-hoc tests (**p* < 0.05, compared with “BMP4+FGF4-treated cells”).

**Figure S3 Hepatoblast differentiation of human ES cells was promoted by FGF removal from differentiation medium as well as human iPS cells**

Human ES cells (KhES-3 cells) were differentiated into the definitive endoderm cells. The definitive endoderm cells were differentiated into hepatoblast-like cells as described in **Figure 1a**. (**a**) The percentages of hepatoblast markers (CK19 and EpCAM)-positive cells were examined by FACS analysis. (**b**) The protein expression levels of hepatoblast markers (CK19 (green)) were examined by immunocytochemical analysis. Nuclei were counterstained with DAPI (blue). The scale bars represent 50 µm. Mean ± SD (*n*=3). Statistical significance was evaluated by one-way ANOVA followed by Tukey’s post-hoc tests to compare all groups. Groups that do not share the same letter are significantly different from each other (**p* < 0.05).

**Figure S4 Cell proliferation was promoted by differentiation with BMP4**

Human iPS cells (YOW-iPS cells) were differentiated into the definitive endoderm cells. The definitive endoderm cells were treated with BMP4+FGF4 (B4+F4), without BMP4 and FGF4 (w/o BF), and with BMP4 (B4) for 5 days. Cell proliferation assay was performed using WST-8. Mean ± SD (*n*=3). Statistical significance was evaluated by one-way ANOVA followed by Tukey’s post-hoc tests to compare all groups. Groups that do not share the same letter are significantly different from each other (**p* < 0.05).

**Figure S5 The effect of FGF removal on hepatic function of the HLCs**

Human ES cells (KhES-3 cells) were differentiated into the definitive endoderm cells. The definitive endoderm cells were differentiated into hepatoblast-like cells as described in **Figure 1a**. The hepatoblast-like cells were differentiated into HLCs. (**a, b**) The ALB (**a**) and urea (**b**) secretion capacities were examined. PHH: Primary human hepatocyte (PHH) cultured for 48hr after plating. (**c**) The protein expression levels of hepatocyte marker, AAT (green), were examined by immunocytochemical analysis. Nuclei were counterstained with DAPI (blue). The scale bars represent 50 µm. (**d**) The percentage of hepatocyte marker (AAT)-positive cells was measured by FACS analysis. Mean ± SD (*n*=3). Statistical significance was evaluated by unpaired two-tail Student’s *t* test (**p* < 0.05, compared with “BMP4+FGF4”).

**Supplemental tables**

**Table S1 The primers used for real-time RT-PCR**

|  | for real time RT-PCR |
| --- | --- |
| Gene Symbol | Primers (forward/reverse; 5' to 3') |
| *AFP* | TGGGACCCGAACTTTCCA/GGCCACATCCAGGACTAGTTTC |
| *CK7* | AGACGGAGTTGACAGAGCTG/GGATGGCCCGGTTCATCTC |
| *FGF1* | ACACCGACGGGCTTTTATACG/CCCATTCTTCTTGAGGCCAAC |
| *FGF2* | AGTGTGTGCTAACCGTTACCT/ACTGCCCAGTTCGTTTCAGTG |
| *FGF4* | CTCGCCCTTCTTCACCGATG/GTAGGACTCGTAGGCGTTGTA |
| *FGF7* | TCCTGCCAACTTTGCTCTACA/CAGGGCTGGAACAGTTCACAT |
| *FGF10* | CATGTGCGGAGCTACAATCAC/CAGGATGCTGTACGGGCAG |
| *FGFR1* | CCCGTAGCTCCATATTGGACA/TTTGCCATTTTTCAACCAGCG |
| *FGFR2* | GGTGGCTGAAAAACGGGAAG/AGATGGGACCACACTTTCCATA |
| *FGFR3* | CCCAAATGGGAGCTGTCTCG/CCCGGTCCTTGTCAATGCC |
| *FGFR4* | CCATAGGGACCCCTCGAATAG/CAGCGGAACTTGACGGTGT |
| *OCT3/4* | GGTTCTCGATACTGGTTCGC/GTGGAGGAAGCTGACAACAA |
| *SOX17* | GTGGACCGCACGGAATTTG/GAGGCCCATCTCAGGCTTG |
| *GAPDH* | GGTGGTCTCCTCTGACTTCAACA/GTGGTCGTTGAGGGCAATG |

**Table S2 The antibodies used for Immunocytochemistry**

| Antigen | Type | Company | Catalog number | Dilution factor |
| --- | --- | --- | --- | --- |
| CK19 | rabbit | abcam | ab52625 | 1:200 |
| HNF4α | mouse | Santa Cruz Biotechnology | sc-374229 | 1:200 |
| AAT | rabbit | Dako | A0012 | 1:200 |
| Alexa Fluor 488 anti-rabbit IgG | donkey | Thermo Fisher Scientific | A21206 | 1:1000 |
| Alexa Fluor 594 anti-mouse IgG | donkey | Thermo Fisher Scientific | A21203 | 1:1000 |

**Table S3 The antibodies used for FACS analysis**

| Antigen | Type | Company | Catalog number | Dilution factor |
| --- | --- | --- | --- | --- |
| CK19 | rabbit | Abcam | ab52625 | 1:200 |
| AFP | rabbit | Dako | sc-8399 | 1:200 |
| EpCAM | mouse | Militenyi Biotec | 130-091-254 | 1:100 |
| Ki67 | rabbit | Abcam | ab15580 | 1:200 |
| SOX17 | mouse | R&D systems | MAB1942 | 1:250 |
| AAT | rabbit | Dako | A0012 | 1:200 |
| Alexa Fluor 488 anti-rabbit IgG | donkey | Thermo Fisher Scientific | A21206 | 1:1000 |
| Alexa Fluor 488 anti-mouse IgG | donkey | Thermo Fisher Scientific | A21202 | 1:1000 |
